# The effect of IL-2 stimulation and treatment of TRPM3 on channel co-localisation with PIP_2_ and NK cell function in myalgic encephalomyelitis/chronic fatigue syndrome patients

**DOI:** 10.1186/s12967-021-02974-4

**Published:** 2021-07-15

**Authors:** Natalie Eaton-Fitch, Hélène Cabanas, Stanley du Preez, Donald Staines, Sonya Marshall-Gradisnik

**Affiliations:** 1 School of Medical Sciences, Griffith University, Gold Coast, Australia; 2 National Centre for Neuroimmunology and Emerging Diseases, Menzies Health Institute Queensland, Griffith University, Gold Coast, Australia; 3 Consortium Health International for Myalgic Encephalomyelitis, Griffith University, Gold Coast, Australia

**Keywords:** Myalgic Encephalomyelitis, Chronic Fatigue Syndrome, Natural killer cells, Transient receptor potential melastatin 3, IL-2, PIP_2_

## Abstract

**Background:**

Myalgic Encephalomyelitis/Chronic Fatigue Syndrome (ME/CFS) is a serious multifactorial disorder. The origin remains ambiguous, however reduced natural killer (NK) cell cytotoxicity is a consistent immunological feature of ME/CFS. Impaired transient receptor potential melastatin 3 (TRPM3), a phosphatidylinositol dependent channel, and impaired calcium mobilisation have been implicated in ME/CFS pathology. This investigation aimed to examine the localisation of TRPM3 at the NK cell plasma membrane and co-localisation with phosphatidylinositol 4,5-bisphosphate (PIP_2_). The effect of IL-2 priming and treatment using pregnenolone sulfate (PregS) and ononetin on TRPM3 co-localisation and NK cell cytotoxicity in ME/CFS patients and healthy controls (HC) was also investigated.

**Methods:**

NK cells were isolated from 15 ME/CFS patients and 15 age- and sex-matched HC. Immunofluorescent technique was used to determine co-localisation of TRPM3 with the NK cell membrane and with PIP_2_ of ME/CFS patients and HC. Flow cytometry was used to determine NK cell cytotoxicity. Following IL-2 stimulation and treatment with PregS and ononetin changes in co-localisation and NK cell cytotoxicity were measured.

**Results:**

Overnight treatment of NK cells with PregS and ononetin resulted in reduced co-localisation of TRPM3 with PIP_2_ and actin in HC. Co-localisation of TRPM3 with PIP_2_ in NK cells was significantly reduced in ME/CFS patients compared with HC following priming with IL-2. A significant increase in co-localisation of TRPM3 with PIP_2_ was reported following overnight treatment with ononetin within ME/CFS patients and between groups. Baseline NK cell cytotoxicity was significantly reduced in ME/CFS patients; however, no changes were observed following overnight incubation with IL-2, PregS and ononetin between HC and ME/CFS patients. IL-2 stimulation significantly enhanced NK cell cytotoxicity in HC and ME/CFS patients.

**Conclusion:**

Significant changes in co-localisation suggest PIP_2_-dependent TRPM3 function may be impaired in ME/CFS patients. Stimulation of NK cells with IL-2 significantly enhanced cytotoxic function in ME/CFS patients demonstrating normal function compared with HC. A crosstalk exists between IL-2 and TRPM3 intracellular signalling pathways which are dependent on Ca^2+^ influx and PIP_2_. While IL-2R responds to IL-2 binding in vitro, Ca^2+^ dysregulation and impaired intracellular signalling pathways impede NK cell function in ME/CFS patients.

**Supplementary Information:**

The online version contains supplementary material available at 10.1186/s12967-021-02974-4.

## Background

Myalgic Encephalomyelitis/Chronic Fatigue Syndrome (ME/CFS) is a highly debilitating and multifactorial condition that is of unknown origin [1]. Diagnosis is currently based on the use of case definitions collectively referred to as the Fukuda criteria (1994), Canadian Consensus Criteria (CCC) (2003) and the International Consensus Criteria (ICC) (2011) [2–4]. Under these criteria a case of ME/CFS is diagnosed by the concurrent presence of symptoms within categories of post-exertional neuroimmune exhaustion, neurological, cardiovascular, autonomic, and neuroendocrine manifestations. While the aetiology remains elusive, ME/CFS may be described as a transient receptor potential (TRP) channelopathy with recent investigations having reported impaired TRPM3 (melastatin) ion channel function [5–8].

TRPM3 belongs to a superfamily of TRP ion channels that are widely expressed in a variety of cells and tissues including the sensory ganglia, central nervous system (CNS), pancreatic beta islets, cardiovascular cells, skeletal muscle cells, genitourinary and immune cells [7, 9]. Due to their widespread expression in the body and their role in biological pathways, TRP ion channel dysfunction is implicated in diverse pathological states that may be categorised as channelopathies. [10, 11]. In 2016, five single nucleotide polymorphisms (SNPs) were identified within the *TRPM3* gene (rs6560200, rs1106948, rs12350232, rs11142822, rs1891301) in natural killer (NK) cells from ME/CFS patients [6]. Subsequently, flow cytometry experiments revealed a significant reduction in TRPM3 surface expression and calcium (Ca^2+^) mobilisation in NK cells isolated from ME/CFS patients compared with healthy controls (HC) [7, 12]. More recently, electrophysiology investigations reported a significant loss of TRPM3 ion channel function in NK cells from ME/CFS patients compared with HC [5, 13, 14]. Thus, these data highlight that mutations in the *TRPM3* gene and TRPM3 ion channel dysfunction may provide a potential biomarker or therapeutic target for ME/CFS.

TRPM3 acts as a non-selective cation channel permeable to manganese (Mn^2+^), sodium (Na^+^) and magnesium (Mg^2+^), however it possesses higher permeability for Ca^2+^ [15, 16]. The activation of TRPM3 channels results in a transient increase in Ca^2+^ leading to a cascade of events that enhances cell function in both excitable and non-excitable cells [12–14]. Calmodulin (CaM), a Ca^2+^ binding protein located at the N-terminus of TRPM3 senses changes in intracellular Ca^2+^ concentration ([Ca^2+^]_I_) to either up- or down-regulate TRPM3 activity [17, 18]. Multiple classes of stimuli enhance TRPM3 function including cell swelling, natural chemicals, toxins and synthetic compounds [16, 19, 20]. For example, pregnenolone sulfate (PregS), an endogenous neurosteroid, has been reported to stimulate TRPM3 ion channel activity (EC_50_ = 12–32 μM) [21] while ononetin rapidly and reversibly inhibits PregS-evoked ionic currents (IC_50_ = 0.2–2 μM) [22].

TRPM3 is also regulated by the recruitment of signalling proteins such as phosphatidylinositol 4,5-bisphosphate (PIP_2_) [23]. PIP_2_ represents less than 1% of membrane phospholipids, but is considered a pleiotropic regulator and key modulator of numerous fundamental cellular processes including ion channel activation [24]. Specifically, upon the activation of phospholipase C (PLC), changes in PIP_2_ lead to the formation of inositol 1,4,5-trisphosphate (IP_3_) and diacylglycerol (DAG) [25]. IP_3_ acts as a secondary messenger whereby upon interaction with its receptor (IP_3_R), on the endoplasmic reticulum (ER), releases Ca^2+^ into the cytosol [26]. Electrophysiology experiments in HEK293 cells overexpressing TRPM3, have suggested that TRPM3 is reliant on the presence of PIP_2_ and inhibition occurs following PIP_2_ depletion [23]. However, the mechanism for how this regulation is achieved is poorly understood as the binding site of PIP_2_ on TRPM3 remains unknown and structural rearrangement of the channel upon PIP_2_ binding is yet to be identified [18, 27, 28].

Lymphocytes, and more specifically NK cells, rely on long-term, sustained Ca^2+^ influx to drive interactions with target antigen peptides which lead to effector functions such as cytotoxicity or cytokine production [29]. Specifically, Ca^2+^ is vital for NK cell cytotoxic function by enabling microtubule rearrangement leading to cytolytic granule polarisation, release of lytic proteins, formation of the perforin pore and granzyme-dependent target cell death [30, 31]. Disturbances in Ca^2+^ homeostasis in lymphocytes can negatively impact immune cell functions and consequently facilitate immune diseases and immunodeficiencies [32]. Meanwhile, the involvement of Ca^2+^ in interleukin-2 (IL-2) signalling, expression and production have been elucidated [33]. IL-2 is known to rapidly enhance NK cell interaction with target cells and enhance cytotoxic activity when the response has previously been weak [34]. Additionally, binding of IL-2 to its receptor promotes enzyme activation of Janus tyrosine kinase (JAK) 1 and 3, and induces multiple pathways such as mitogen activated protein kinase (MAPK), phosphoinositide 3-kinase (PI3K) and signal transducer and activator of transcription (STAT) [35]. Reduced NK cell cytotoxicity has been consistently reported in ME/CFS compared with HC [36–40]. Therefore, these findings suggest that impaired NK cell cytotoxicity is a reliable and appropriate cellular model for continued research on dysregulated Ca^2+^ signalling and impaired TRPM3 ion channel function to elucidate the pathomechanism of ME/CFS [41].

The aim of this investigation was to characterise, for the first time, the co-localisation of TRPM3 with the Ca^2+^-dependent regulator, PIP_2_, using immunofluorescent technique. Following overnight treatment of NK cells with PregS and ononetin, the implications of TRPM3 activation or inhibition on co-localisation with PIP_2_ was determined. Furthermore, to investigate whether NK cell cytotoxicity is impaired due to TRPM3 dysfunction, the effects of overnight IL-2 stimulation and treatment using PregS and ononetin on NK cell cytotoxicity was characterised using flow cytometry. This current investigation is novel as it provides insight into the potential role of Ca^2+^- and PIP_2_-dependent IL-2 and TRPM3 cellular pathways and the effect on NK cell function.

## Methods

### Recruitment

ME/CFS patients and HC were contacted using the National Centre for Neuroimmunology and Emerging Diseases (NCNED) patient database which consists of approximately 600 participants. Participants were screened in accordance with the CCC and ICC case definitions for ME/CFS using a comprehensive online questionnaire. ME/CFS patients were included if they met CCC, and subsequently the Fukuda, case definitions for diagnosis and reported being diagnosed by a physician. 50 ME/CFS patients from South-East Queensland and Melbourne were determined eligible and were invited to volunteer in this project. Of those contacted, 15 ME/CFS patients responded and agreed to participate. ME/CFS patients were then age- and sex-matched with HC. The HC group were defined as those who have not been diagnosed with any underlying illness and are non-fatigued. All participants were required to be aged between 18 and 60 years, had a BMI between 18.5 and 29.9 (kg/m^2^) and were non-smokers. BMI was determine using the World Health Organisation categories for BMI: underweight < 18.5; normal weight 18.5–24.9; overweight 25.0–29.9; and obese > 30 [42].

Participants were excluded if they reported a history of alcohol abuse, cardiovascular disease, diabetes, metabolic syndrome, thyroid disease, malignancies, insomnia, and if they were pregnant or breastfeeding. Furthermore, all participants were excluded if they reported the use of pharmacological agents that directly or indirectly interfere with TRPM3 ion channel function as well as Ca^2+^ signalling and immune cell activity. Participants were provided with the option to cease any conflicting medications for a minimum of 14 days prior to blood donation with the approval of their physician. This investigation was approved by the Gold Coast Human Research Ethics Committee (HREC/2019/56469) and Griffith University Human Research Ethics Committee (GU/2019/1005).

### Participant data collection and sample collection

All participants completed an online questionnaire to provide sociodemographic background, medical history, medications, and symptom history for ME/CFS patients. ME/CFS symptom survey responses were grouped into 10 symptom categories including: (i) cognitive difficulties (slowed thought, impaired concentration and memory consolidation issues); (ii) pain (headaches, muscle pain and multi-joint pain); (iii) sleep disturbances (reversed sleep cycle, disturbed sleep cycle, unrefreshing sleep); (iv) sensory disturbances (sensitivity to touch, vibration, taste, odour and sound, poor coordination or balance); (v) immune disturbances (flu-like symptoms, sore throat, tender lymph nodes); (vi) gastrointestinal disturbances (abdominal pain, nausea, bloating); (vii) cardiovascular disturbances (orthostatic intolerances, light headedness, heart palpitations); (viii) respiratory disturbances (difficulty breathing and air hunger); (ix) thermostatic instability (abnormal sweating episodes, hot flushes and cold extremities); and (x) urinary disturbances (changed urination frequency and painful bladder). The 36-item short form health survey (SF-36) and World Health Organization (WHO) Disability Assessment Schedule (DAS) were used to determine level of disability and quality of life [43, 44].

After obtaining written consent, a total of 85 ml of whole, non-fasted blood was collected into ethylenediaminetetraacetic acid (EDTA) tubes via venepuncture by a qualified phlebotomist from each participant between 7:00am and 11:00am at collection locations including Royal Melbourne Hospital, Griffith University, Royal Brisbane and Women’s Hospital, Robina Hospital, Toowoomba Base Hospital, Sunshine Coast University Hospital and Tweed Hospital. Five ml of EDTA whole blood was used for red blood cell count, white blood cell count and granulocyte cell count within 4 h of blood collection for each participant.

### Peripheral blood mononuclear cell and natural killer cell isolation

Samples were delivered to the laboratory de-identified using a unique code by an independent blood collector. Eighty ml of blood was used for peripheral blood mononuclear cells (PBMC) isolation by density gradient centrifugation using Ficoll (GE Healthcare, Uppsala, Sweden) as previously described [45]. Isolated PBMCs were adjusted to a final concentration of 5 × 10^7^ cells/ml for NK cell isolation.

NK cells were isolated by immunomagnetic selection using the EasySep Negative Human NK cell Enrichment Kit (Stem Cell Technologies, Vancouver, BC, Canada). NK cell purity was defined by CD3^−^CD56^+^ surface expression using flow cytometry (Additional file 1: Fig. S1). Specifically, NK cells were incubated for 20 min at room temperature in the presence of CD3 PE-Cy7 (5 µl/test) and CD56 APC (20 µl/test) monoclonal antibodies (Becton Dickinson [BD] Biosciences, San Jose, CA, USA). Cells were acquired at 10,000 events using the Accuri C6 flow cytometer (BD Biosciences, San Diego, CA, USA). Acceptable NK cell purity was ≥ 90% (Additional file 1: Fig. S2).

### Interleukin-2 stimulation and in vitro drug treatment

Freshly isolated NK cells (4.5 × 10^6^ cells) were stimulated with 20 IU/ml of recombinant human IL-2 (specific activity 5 × 10^6^ IU/mg) (Miltenyi Biotech, BG, Germany). NK cells (7.5 × 10^5^ cells/condition) were also treated with the following drug combinations: (i) 30 µM PregS (Tocris Bioscience, Bristol, UK); (ii) 30 µM PregS and 3 µM Ononetin (Tocris Bioscience, Bristol, UK). These drug concentrations were determined using dose response analysis (Additional file 1: Figs. S3 and S4). NK cells were stimulated with IL-2 and treated with the above-mentioned drug combinations for 24 h at 37 °C with 5% CO_2_ in Roswell Park Memorial Institute Medium (RPMI)-1640 (Invitrogen Life Technologies, Carlsbad, CA, USA) supplemented with 10% fetal bovine serum (FBS) (Invitrogen Life Technologies, Carlsbad, CA, USA). The treatment of NK cells with PregS and Ononetin for 24 h was to correspond with recommended duration of pre-activation of NK cells by cytokines.

### Immunofluorescence

Confocal microscopy imaging techniques were used to determine co-localisation of TRPM3 (Alomone, Jerusalem, Israel) with PIP_2_ and actin (Abcam, Cambridge, UK). Freshly isolated NK cells were immobilized on Corning® Cell-Tak™ Cell and Tissue Adhesive coated coverslips (BD Biosciences, San Jose, CA, USA). Fixation was completed using phosphate-buffered saline (PBS) 1X (Invitrogen Life Technologies, Carlsbad, CA, USA) with 3% Paraformaldehyde (Sigma-Aldrich, St. Louis, MO, USA) for 20 min at room temperature (RT). NK cells were permeabilised using PBS 1X + 0.02% Tween 20 (Sigma-Aldrich, St. Louis, MO, USA) for 30 min at RT. Non-specific staining was blocked for 1 h using 3% bovine serum albumin (BSA) (Sigma-Aldrich, St. Louis, MO, USA) in PBS 1X at RT. NK cells were incubated overnight (16 h) at 4 °C with primary antibodies for TRPM3 (1:6,000) (Alomone, Jerusalem, Israel) and PIP_2_ (1:3,000) (Abcam, Cambridge, UK) in PBS 1X + 3% BSA. NK cells were incubated with secondary antibodies for 30 min at RT protected from light (anti-Mouse Alexa Fluor 594 [1:2,000] and anti-Rabbit Alexa Fluor 488 [1:2,000]) in PBS 1X + 3% BSA. Phalloidin Alexa Fluor 647 (1:500) (Invitrogen, Massachusetts, USA) was added for 1 h at RT. The adenine–thymine base pairs were stained using DAPI (300 nM) (Invitrogen Life Technologies, Carlsbad, CA, USA) for 5 min at RT. Following each step, NK cells were washed three times with sterile PBS 1X for five minutes. The final wash was completed with distilled water. PIP_2_ immunofluorescence was confirmed by using non-permeabilised cells while TRPM3 immunofluorescence was confirmed using the corresponding blocking peptide (Alomone, Jerusalem, Israel). Fluorescence was observed using an inverted confocal microscope, Nikon A1R (Nikon, NIS-Elements V5.2, Tokyo, Japan), with a 60 × oil immersion objective. Co-localisation of TRPM3, PIP_2_ and actin was assessed by NIS-Elements Advanced Research software (Nikon, NIS-Elements V5.2, Tokyo, Japan) by measuring the Pearson’s correlation coefficient (PCC), Mander’s overlap coefficient (MOC) and K1/K2 co-localisation coefficients. Specifically, PCC was used to measure co-variance between two colours (+ 1 [positive correlation] and − 1 [negative correlation]). While MOC ranges from 0 to + 1 to represent the percentage of pixels which overlap. K1 and K2 coefficients were derived from MOC to separately represent the fraction of total fluorescence by two channels.

### Natural killer cell cytotoxic activity

NK cell cytotoxicity was conducted as previously described methods [46]. NK cells were labelled with Paul Karl Horan (PKH)-26 (3.5 µl/test) for 5 min (Sigma-Aldrich, St. Louis. MO, USA) and incubated with K562 cells for 4 h at 37 °C with 5% CO_2_ in RPMI-1640 supplemented with 10% FBS. NK cells and target K562 cells were combined at effector to target (E:T) ratio of 12.5:1 and 6.25:1 while K562 alone was plated as a control. Following incubation for 4 h, cells were stained using Annexin V (2.5 µl/test) and 7-amino-actinomycin (7-AAD) (2.5 µl/test) (BD Biosciences, San Jose, CA, USA). Cytotoxic activity was determined by measuring K562 cell death using the Accuri C6 flow cytometer (Additional file 1: Fig. S5). 20,000 events were recorded for each experiment. For the two ratios, the percentage of target cell lysis was calculated as previously described [46] and outlined below:

$$\mathrm{Cytotoxicity }\left(\mathrm{\%}\right)= \frac{(\mathrm{early stage apoptosis}+\mathrm{late stage apoptosis}+\mathrm{necrotic cells}}{\mathrm{All K}562\mathrm{ cell events}}\times 100$$

### Biological and chemical reagents

PregS (product code: RDS537650) and ononetin (product code: RDS514350) were purchased from In Vitro Technologies. PregS and ononetin were prepared at 100 mM stock solution in 100% DMSO for up to one month. Human, premium grade IL-2 was purchased from Miltenyi Biotechnologies (product code: 130–097-744) stored at 100,000 IU stock in distilled water for up to one month. Flow cytometry antibodies were purchased from BD Biosciences, CD3 PE-Cy7 (product code: 563423), CD56 APC (product code: 555518), 7-AAD (product code: 559925) and Annexin V (product code: 550474) were stored and used following manufacturers recommendations. Anti-PIP_2_ was purchased from Abcam (product code: ab11039) while anti-TRPM3 was purchased from Alomone Labs (Product code: ACC-050) and reconstituted at 0.8 mg/ml in distilled water. Secondary antibodies: goat anti-rabbit alexa fluor 488 (product code: A1034) and goat anti-mouse alexa fluor 594 (product code: A11032) were purchased from Thermofisher.

### Statistical analysis

Visual and computed methods were used to determine normality of independent data. Histogram plots and Shapiro–Wilk test were used to assess normality of distribution of investigated parameters. Differences were analysed by Mann–Whitney U non-parametric T test or independent samples T test depending on normality. PCC, MOC and K1/K2 co-localisation coefficient were used to analyse the co-localisation between TRPM3, PIP_2_ and actin. The values p < 0.05 were considered statistically significant. Flow cytometry data was exported from Accuri C6 software and confocal data was analysed using NIS-Elements Advanced Research version 5.2. Statistical analysis was done using GraphPad Prism V8 (GraphPad Software Inc., Version 8, La Jolla, CA, USA) and SPSS v26 (IBM Corp, USA). Data were presented as mean ± standard error of mean (SEM) unless otherwise stated. Significance was set at p < 0.05.

## Results

### Participant and disease characteristics

During the study period of December 2019 to December 2020, 15 ME/CFS patients and 15 age- and sex-matched HC participated in this project. All ME/CFS patients reported symptoms fulfilling the CCC and no other fatigue related illness that may account for their symptoms. Table 1 includes demographic data of the participants. The mean BMI of HC were within normal range (18.5–24.9) while ME/CFS patients were slightly elevated (24.95 ± 1.07).

**Table 1:** Participant demographics

	HC	ME/CFS	P-value
Age (years)	44.2 ± 3.14	43.93 ± 2.94	0.951
Gender n (%)			
Female	9 (60%)	9 (60%)	1.000
Male	6 (40%)	6 (40%)	
BMI (kg/m^2^)	23.8 ± 0.67	24.95 ± 1.07	0.374
Work status			
Full time	10 (66.7%)	3 (20%)	** < 0.001**
Part time	4 (26.7%)	3 (20%)	
Casual	0 (0%)	1 (6.7%)	
Unemployed	1 (6.7%)	2 (13.3%)	
Illness/disability	0 (0%)	6 (40%)	
Education			
Primary education	0 (0%)	0 (0%)	0.751
High school	1 (6.7%)	3 (20%)	
Undergraduate	5 (33.3%)	6 (40%)	
Postgraduate/doctoral	7 (46.7%)	5 (33.3%)	
Other	2 (13.3%)	1 (6.7%)	

Values in bold indicate statistical significance

*HC* healthy controls, *ME* Myalgic encephalomyelitis, *CFS* chronic fatigue syndrome, *BMI* body mass index

The SF-36 and WHO DAS surveys were used to assess quality of life (QoL) in ME/CFS patients compared with HC. As reported in Table 2, mean scores were significantly reduced in ME/CFS patients across all SF-36 domains excluding limitations due to emotional role. Lowest scores were reported in ME/CFS patients for the limitations due to physical role domain (28.33 ± 6.87). Mean scores obtained from the WHO DAS survey report a significant increase in ME/CFS patient disability scores across all domains. Highest disability scores were recorded for the life activities domain (56.25 ± 8.09). Comparison of blood parameters between ME/CFS patients and HC found no significant differences other than eosinophil count, however individual blood parameters were within normal range according to Queensland Health Pathology and Victorian Pathology.

**Table 2:** Participant quality of life, disability scores and serology

	HC	ME/CFS	P-value
SF-36 (%)			
Physical functioning	96.33 ± 2.89	44.00 ± 7.24	** < 0.001**
Physical role	93.33 ± 2.89	28.33 ± 6.87	** < 0.001**
Pain	89.33 ± 3.022	42.833 ± 6.70	** < 0.001**
General health	79.43 ± 3.48	34.71 ± 4.64	** < 0.001**
Social functioning	94.16 ± 2.95	34.17 ± 6.03	** < 0.001**
Emotional role	93.33 ± 3.02	70.00 ± 6.44	**0.007**
Emotional wellbeing	74.07 ± 4.48	43.82 ± 3.92	** < 0.001**
WHO DAS (%)			
Communication & understanding	7.19 ± 1.96	40.81 ± 4.49	** < 0.001**
Mobility	2.67 ± 0.83	40.67 ± 7.44	** < 0.001**
Self-care	0 ± 0	20.41 ± 5.37	**0.004**
Interpersonal relationships	7.92 ± 2.69	32.91 ± 4.97	** < 0.001**
Life activities	6.67 ± 2.23	56.25 ± 8.09	** < 0.001**
Participation in society	3.12 ± 1.55	50.62 ± 5.86	** < 0.001**
Full blood count			
White cell count (× 10^9^/L)	5.40 ± 0.32	6.07 ± 0.48	0.262
Lymphocytes (× 10^9^/L)	1.76 ± 0.15	1.88 ± 0.11	0.517
Neutrophils (× 10^9^/L)	3.07 ± 0.26	3.51 ± 0.38	0.345
Monocytes (× 10^9^/L)	0.40 ± 0.03	0.43 ± 0.04	0.598
Eosinophils (× 10^9^/L)	0.12 ± 0.02	0.18 ± 0.02	**0.033**
Basophils (× 10^9^/L)	0.04 ± 0.01	0.04 ± 0.004	0.631
Platelets (× 10^9^/L)	239.67 ± 14.59	254.20 ± 11.10	0.435
Red Cell Count (× 10^12^/L)	4.82 ± 0.13	4.67 ± 0.10	0.356
Haematocrit	0.43 ± 0.01	0.42 ± 0.01	0.806
Haemoglobin (g/L)	143.07 ± 4.10	141.0 ± 2.76	0.680

Values in bold indicate statistical significance

*HC* healthy controls, *ME* Myalgic encephalomyelitis, *CFS* chronic fatigue syndrome, *SF-36* 36-item short form survey, *WHO* world health organization, *DAS* disability assessment schedule

All ME/CFS patients successfully completed the NCNED registry questionnaire that encompasses questions pertaining to the Fukuda, CCC and ICC diagnostic criteria. On average, patients were 29.6 years of age at diagnosis and have experienced symptoms of ME/CFS for 14.93 years (Table 3). Moreover, 86.7% of patients reported an infection prior to onset of symptoms. All ME/CFS patients (100%) reported experiencing symptoms including cognitive difficulties, body pain, sleep disturbances, sensory disturbances, and cardiovascular disturbances (100%). Immune disturbances (93.3%) and gastrointestinal disturbances (93.3%) were consistently reported.

**Table 3:** ME/CFS symptom characteristics

Age of diagnosis (years [mean ± SEM])	29.6 ± 3.05
Disease duration (years [mean ± SEM])	14.93 ± 3.17
Infectious onset, n(%)	13 (86.7%)
Cognitive difficulties	
Yes	15 (100%)
No	0 (0%)
Pain	
Yes	15 (100%)
No	0 (0%)
Sleep disturbances	
Yes	15 (100%)
No	0 (0%)
Sensory disturbances	
Yes	15 (100%)
No	0 (0%)
Immune disturbances	
Yes	14 (93.3%)
No	1 (6.7%)
Gastrointestinal disturbances	
Yes	14 (93.3%)
No	1 (6.7%)
Cardiovascular disturbances	
Yes	15 (100%)
No	0 (0%)
Respiratory disturbances	
Yes	8 (53.3%)
No	7 (46.7%)
Thermostatic instability	
Yes	11 (73.3%)
No	4 (26.7%)
Urinary disturbances	
Yes	5 (33.3%)
No	10 (66.7%)

*ME* Myalgic encephalomyelitis, *CFS* chronic fatigue syndrome, *SEM* standard error of mean, *n* number

### Co-localisation of TRPM3 at the NK cell membrane in HC following modulation with PregS and ononetin

**Fig. 1:**
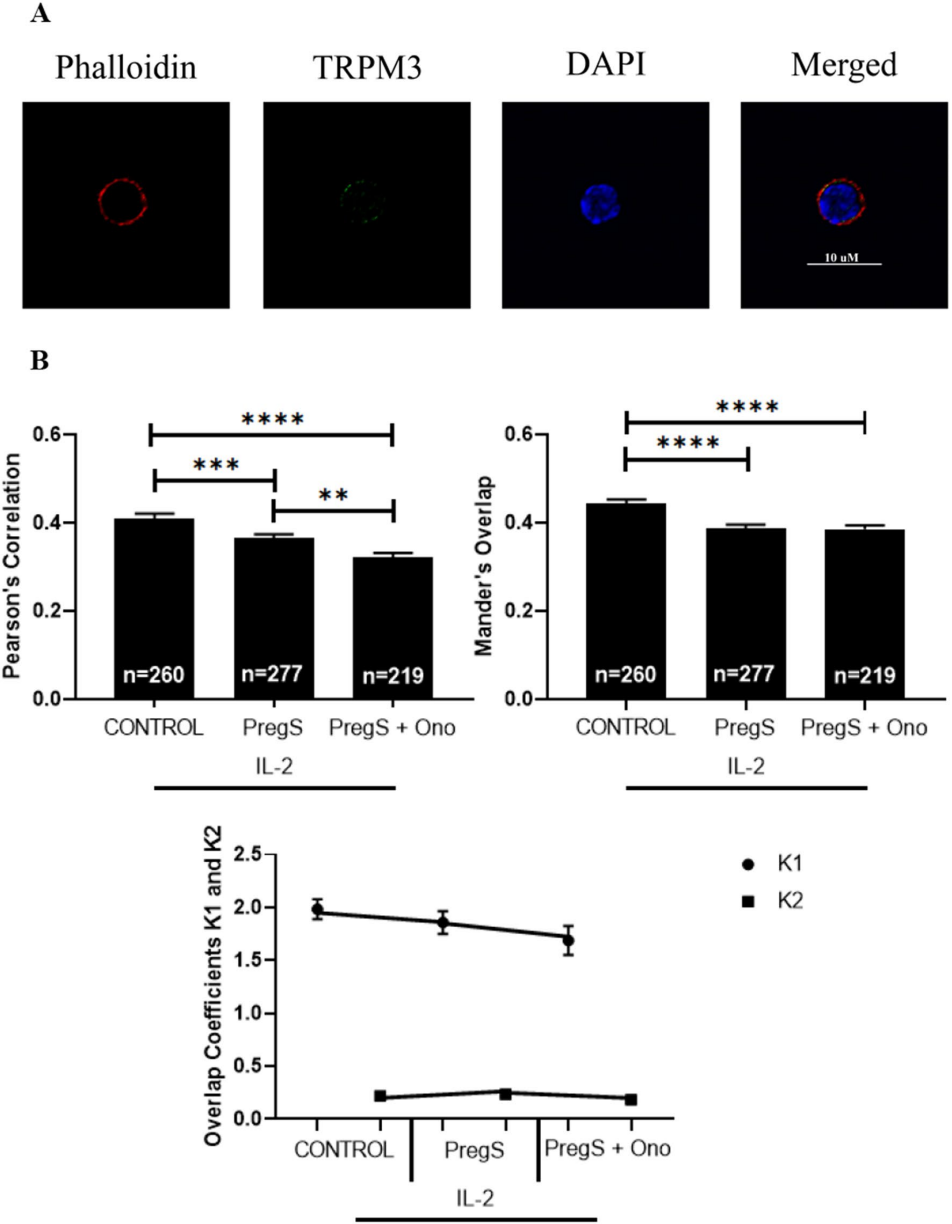
Co-localisation and immunofluorescent images of TRPM3 with actin in NK cells of HC. **A** Example of immunostaining of actin (phalloidin, red), nucleus (DAPI, blue), TRPM3 (green) in an NKLa cell under control IL-2 conditions. Cells were stimulated overnight with IL-2 (20 IU) (control) and treated with PregS (30 µM) and ononetin (3 µM). Images taken using Nikon A1R + confocal microscopy. **B** Bar graphs represent correlation between target antigens using Pearson’s correlation coefficient and the degree of overlap between target antigens using Mander’s overlap coefficient. Co-localisation coefficients K1 (TRPM3) and K2 (actin) were used to determine contribution to co-localisation. Number of cells analysed are included within bar graphs: Control n = 260; PregS n = 277; and PregS + Ono n = 219. Data presented as mean ± SEM and *p < 0.05; **p < 0.01. TRPM3, transient receptor potential Melastatin; PregS, pregnenolone sulfate; Ono, ononetin; SEM, standard error of mean; IL-2, interleukin 2

Confocal immunofluorescent technique was used to visualise TRPM3 at the NK cell membrane. Quantitative co-localisation values demonstrated a weak to moderate degree of co-localisation (MOC > 0.3 and < 0.5) of TRPM3 with actin of the NK cell membrane in HC (Fig. 1A). There was a significant reduction in the overlap, or co-localisation of TRPM3 with the plasma membrane in PregS (30 µM) treated cells (PCC = 0.365, p = 0.007: MOC = 0.428, p=0.0004) and PregS (30 µM) + ononetin (3 µM) treated cells (PCC = 0.323, p =  < 0.0001: MOC = 0.383, p =  < 0.0001) compared with the untreated cells (PCC = 0.409: MOC = 0.471) (Fig. 1B). Moreover, there was a significant reduction in co-localisation of TRPM3 with actin in PregS (30 µM) + ononetin (3 µM) treated cells compared with PregS treated cells (PCC, p = 0.0022: MOC, p = 0.0008).

Using co-localisation coefficients K1 and K2 it showed the impact of TRPM3 contribution (K1) to co-localisation in comparison with actin (K2). The highest contribution to co-localisation by TRPM3 was reported in the control condition (K1 = 1.98). There was a slight decline reported in TRPM3 contribution to co-localisation following treatment with PregS (K1 = 1.859) and PregS + ononetin (K1 = 1.688) (Fig. 1B). The contribution to co-localisation by actin fluorescence was similar between the control (K2 = 0.220) and PregS (30 µM) condition (K2 = 0.237) with a decline following PregS (30 µM) + ononetin (3 µM) treatment (K2 = 0.183).

### Co-localisation of TRPM3 at the NK cell membrane in ME/CFS patients following modulation with PregS and ononetin

**Fig. 2:**
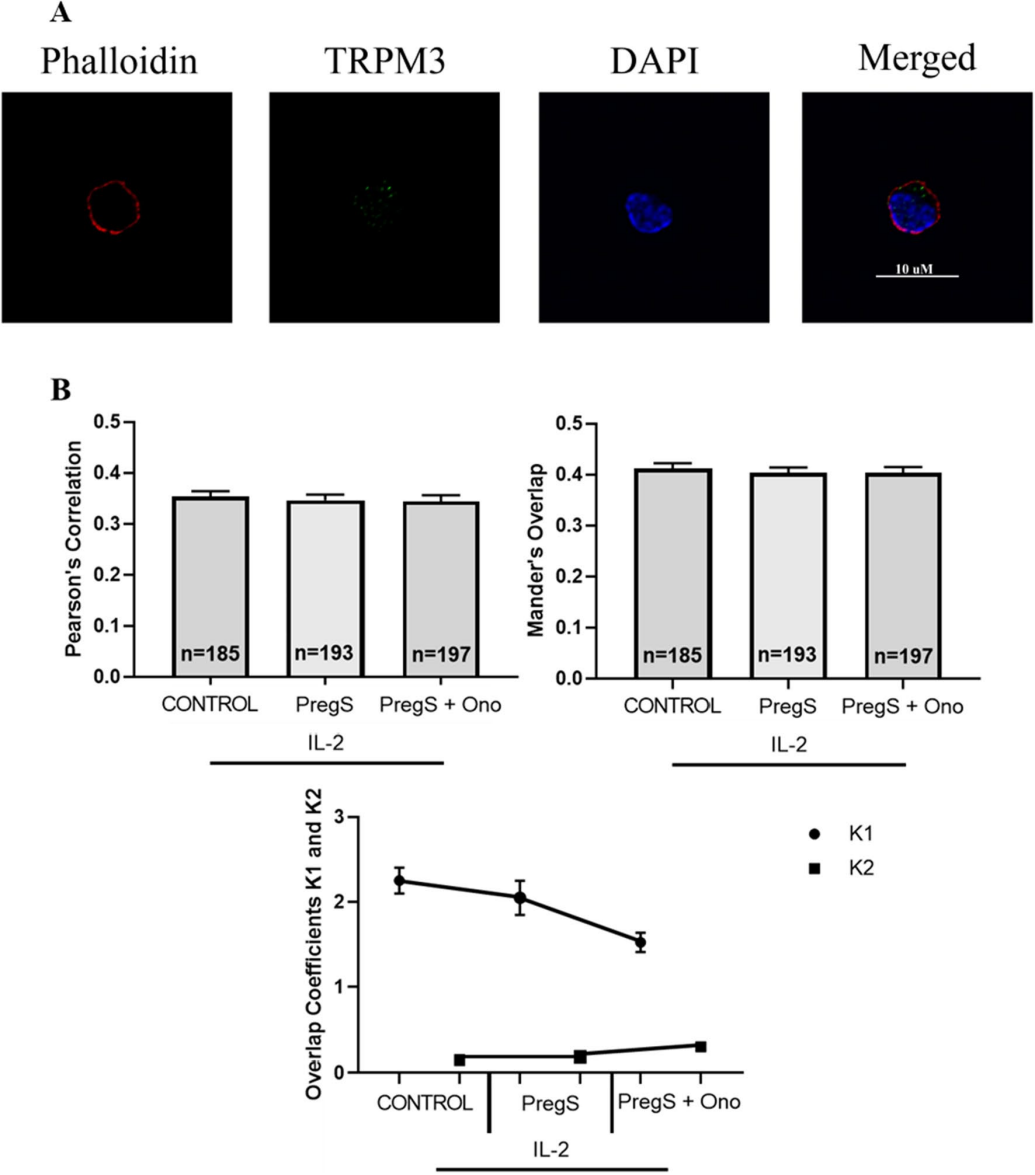
Co-localisation and immunofluorescent images of TRPM3 with actin in NK cells of ME/CFS patients. **A** Example of immunostaining of actin (phalloidin, red), nucleus (DAPI, blue), TRPM3 (green) in NK cell under control IL-2 condition. Cells were stimulated overnight with IL-2 (20 IU) (control) and treated with PregS (30 µM) and ononetin (3 µM). Images taken using Nikon A1R + confocal microscopy. **B** Bar graphs represent correlation between target antigens using Pearson’s correlation coefficient and the degree of overlap between target antigens using Mander’s overlap coefficient. Co-localisation coefficients K1 (TRPM3) and K2 (actin) were used to determine contribution to co-localisation. Number of cells analysed are included within bar graphs: Control n = 185; PregS n = 193; and PregS + Ono n = 197. Data presented as mean ± SEM. TRPM3, transient receptor potential Melastatin; PregS, pregnenolone sulfate; Ono, ononetin; IL-2, interleukin 2

Quantitative co-localisation values demonstrated a weak to moderate degree of co-localisation (MOC > 0.3 and < 0.5) of TRPM3 with actin at the NK cell membrane in ME/CFS patients (Fig. 2A). There was no significance reported in co-localisation values PCC and MOC for PregS (30 µM) (PCC = 0.346, MOC = 0.403) and PregS (30 µM) + ononetin (3 µM) (PCC = 0.344, MOC = 0.404) treated cells (Fig. 2B).

Inversely, the contribution of TRPM3 to co-localisation declined following PregS (30 µM) (K1 = 2.05) and PregS (30 µM) + ononetin (3 µM) (K1 = 1.52) treatments when compared with control (K1 = 2.25) (Fig. 2B). The contribution to co-localisation by actin fluorescence was similar between the control, (K2 = 0.145) and PregS (30 µM) condition (K2 = 0.84) followed by an incline following PregS (30 µM) and ononetin (3 µM) treatment (K2 = 0.296).

### Co-localisation of TRPM3 with PIP_2_ in healthy controls following modulation with PregS and ononetin

**Fig. 3:**
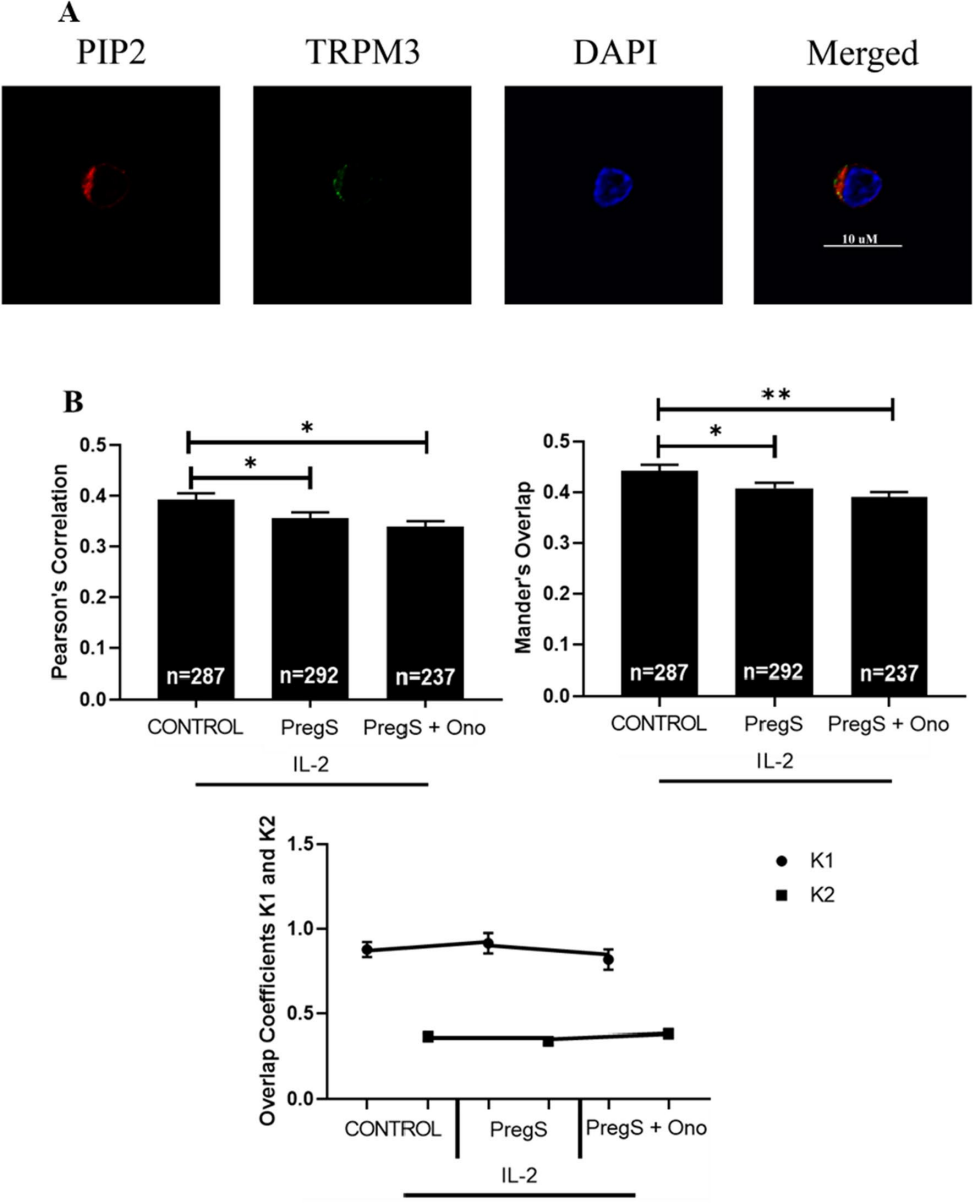
Co-localisation and immunofluorescent images of TRPM3 with PIP_2_ in NK cells of HC. **A** Example of immunostaining of PIP_2_ (red), nucleus (DAPI, blue), TRPM3 (green)) in an NK cell under control IL-2 conditions. Images taken using Nikon A1R + confocal microscopy. **B** Bar graphs represent correlation between target antigens using Pearson’s correlation coefficient and the degree of overlap between target antigens using Mander’s overlap coefficient. Co-localisation coefficients K1 (TRPM3) and K2 (PIP_2_) were used to determine contribution to co-localisation. Number of cells analysed are included within bar graphs: Control n = 287; PregS n = 292; and PregS + Ono n = 237. Data presented as mean ± SEM and *p < 0.05; **p < 0.01. TRPM3, transient receptor potential Melastatin; PIP_2_, phosphatidylinositol 4,5-bisphosphate; PregS, pregnenolone sulfate; Ono, ononetin; IL-2, interleukin 2

Quantitative co-localisation values demonstrated a moderate degree of co-localisation of TRPM3 with PIP_2_ at the NK cell membrane in HC (Fig. 3A). There was a significant reduction in the co-localisation of TRPM3 with PIP_2_ following PregS (30 µM) (PCC = 0.356, p = 0.0465: MOC = 0.408, p = 0.0447) and PregS (30 µM) + ononetin (3 µM) (PCC = 0.337, p = 0.0126: MOC = 0.390, p = 0.0096) treated cells compared with untreated cells (Fig. 3B).

The contribution of TRPM3 to co-localisation yielded similar results following PregS (30 µM) (K1 = 0.914) and PregS (30 µM) + ononetin (3 µM) (K1 = 0.818) treatments when compared with control condition (K1 = 0.877) (Fig. 3B). The contribution to co-localisation by PIP_2_ fluorescence was similar between the control (K2 = 0.365) and PregS (K2 = 0.340) conditions followed by a slight incline following PregS (30 µM) and ononetin (3 µM) treatment (K2 = 0.382).

### Co-localisation of TRPM3 with PIP_2_ in ME/CFS patients following modulation with PregS and ononetin

**Fig. 4:**
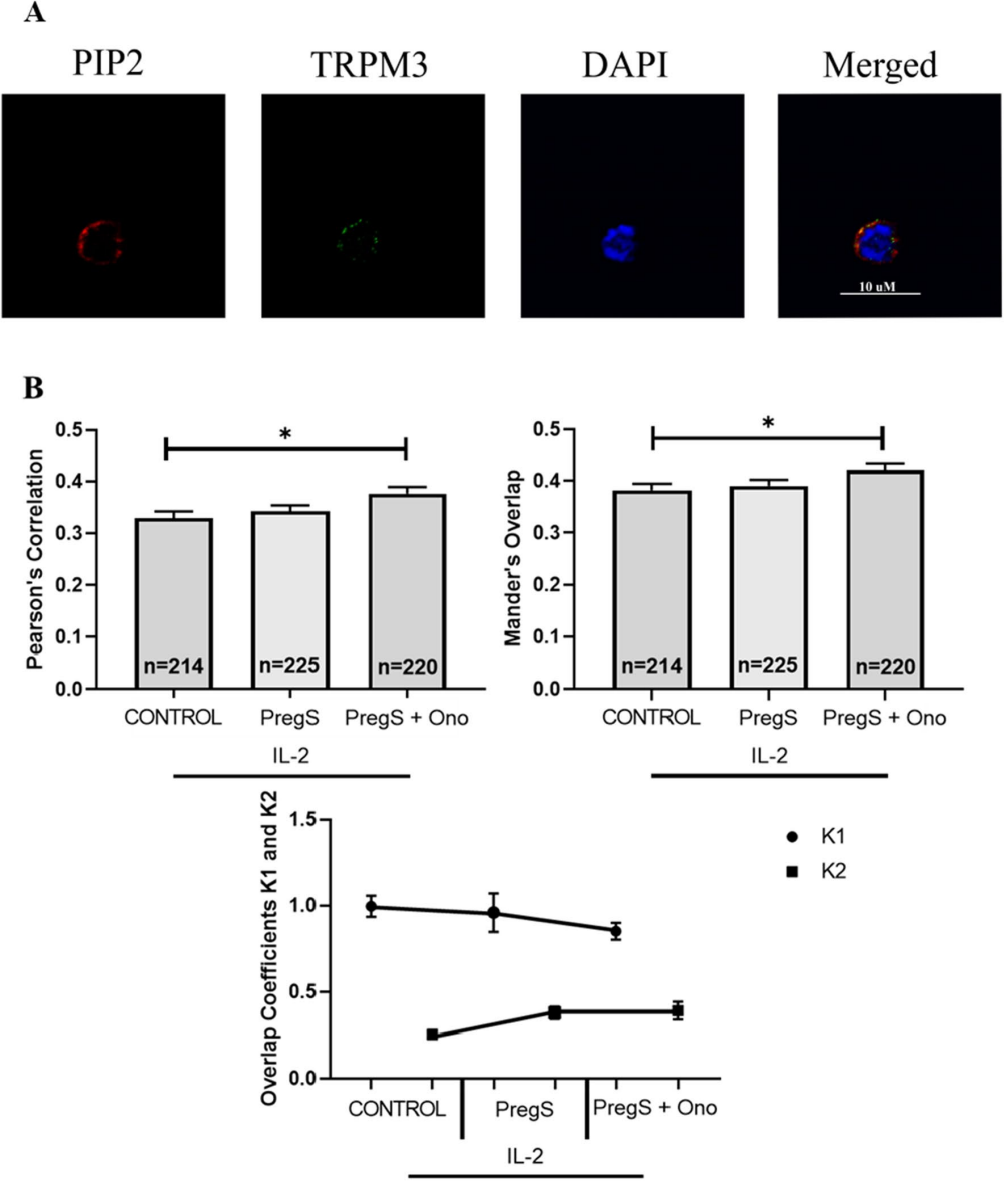
Co-localisation and immunofluorescent images of TRPM3 with PIP_2_ in NK cells of ME/CFS. patients. **A** Example of immunostaining of PIP_2_ (red), nucleus (DAPI, blue), TRPM3 (green) in NK cell under control IL-2 conditions. Cells were stimulated overnight with IL-2 (20 IU) (control) and treated with PregS (30 µM) and ononetin (3 µM). Images taken using Nikon A1R + confocal microscopy. **B** Bar graphs represent correlation between target antigens using Pearson’s correlation coefficient and the degree of overlap between target antigens using Mander’s overlap coefficient. Co-localisation coefficients K1 (TRPM3) and K2 (PIP_2_) were used to determine contribution to co-localisation. Number of cells analysed are included within bar graphs: Control n = 214; PregS n = 225; and PregS + Ono n = 220. Data presented as mean ± SEM and *p < 0.05. TRPM3, transient receptor potential Melastatin; PIP_2_, phosphatidylinositol 4,5-bisphosphate; PregS, pregnenolone sulfate; Ono, ononetin; IL-2, interleukin 2

Quantitative co-localisation values demonstrated a moderate degree of co-localisation of TRPM3 with PIP_2_ at the NK cell membrane in ME/CFS patients (Fig. 4A). There was a significant increase in the co-localisation of TRPM3 with PIP_2_ following PregS (30 µM) + ononetin (3 µM) treated cells (PCC = 0.376, p = 0.0133: MOC = 0.421, p = 0.0364) compared with untreated cells (PCC = 0.329, MOC = 0.382) (Fig. 4B).

The contribution of TRPM3 to co-localisation slightly decreased following PregS (K1 = 0.959) and PregS (30 µM) + ononetin (3 µM) (K1 = 0.850) treatments when compared with control condition (K1 = 0.996) (Fig. 4B). The contribution to co-localisation by PIP_2_ fluorescence increased after PregS (30 µM) (K2 = 0.378) and PregS (30 µM) and ononetin (3 µM) treatment (K2 = 0.390) in comparison with control condition (K2 = 0.253).

### Co-localisation of TRPM3 in ME/CFS compared with HC

**Fig. 5:**
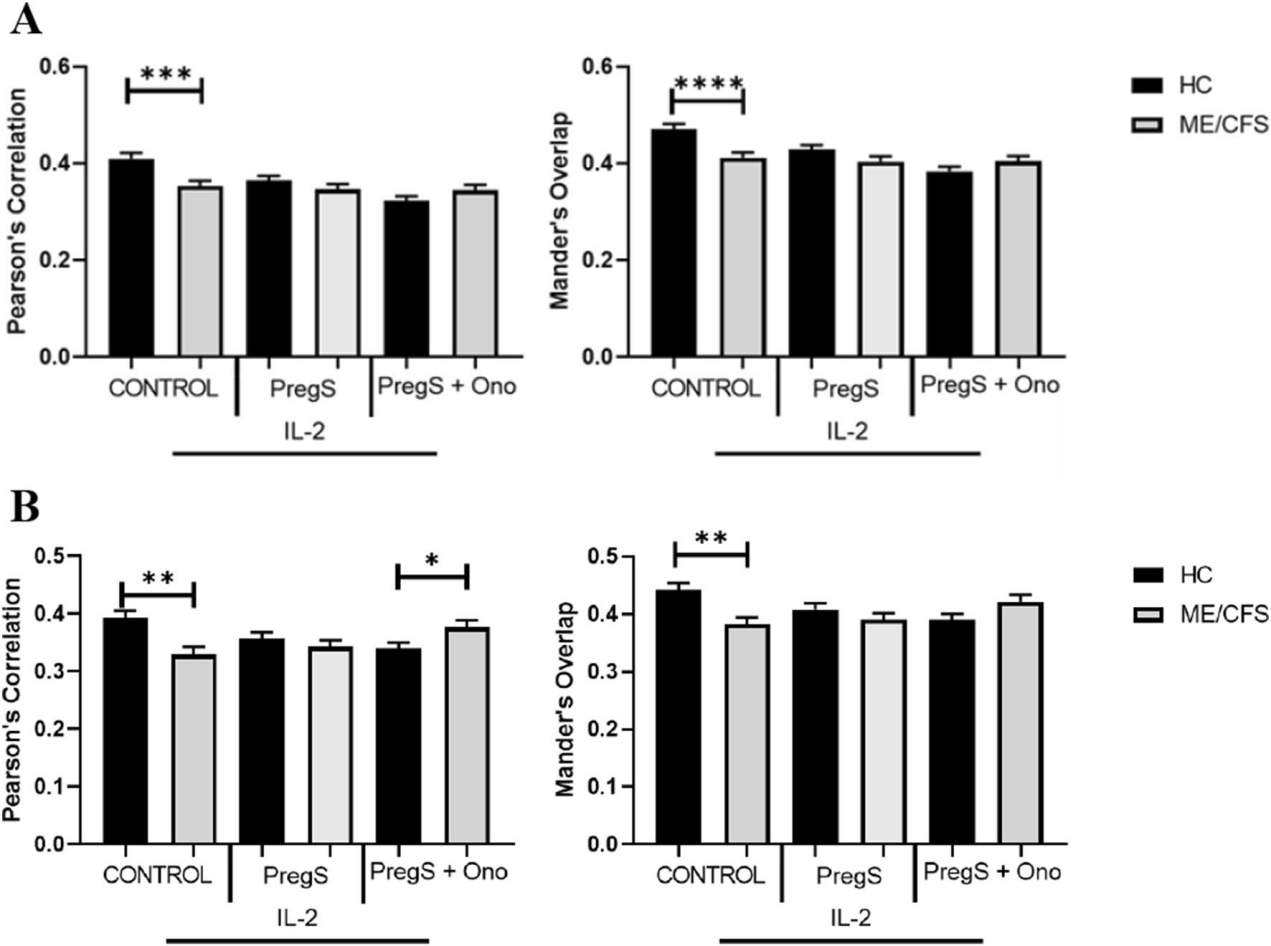
**A** Mann Whitney U test using PCC and MOC co-localisation values of TRPM3 with actin on NK cells of ME/CFS patients and HC. **B** Mann Whitney U test using PCC and MOC co-localisation values of TRPM3 with PIP_2_ on NK cells of ME/CFS patients and HC. Co-localisation values obtained following IL-2 stimulation (20 IU) (control) and pharmacological treatment using PregS (30 µM) and PregS + Ononetin (3 µM). Bar graphs represent correlation between target antigens using PCC represent degree of overlap between target antigens using MOC. Data presented as mean ± SEM and *p < 0.05; **p < 0.01; ***p < 0.001; ****p < 0.0001. HC, healthy control; ME/CFS, Myalgic encephalomyelitis/chronic fatigue syndrome; PregS, pregnenolone sulfate; Ono, ononetin

**Fig. 6:**
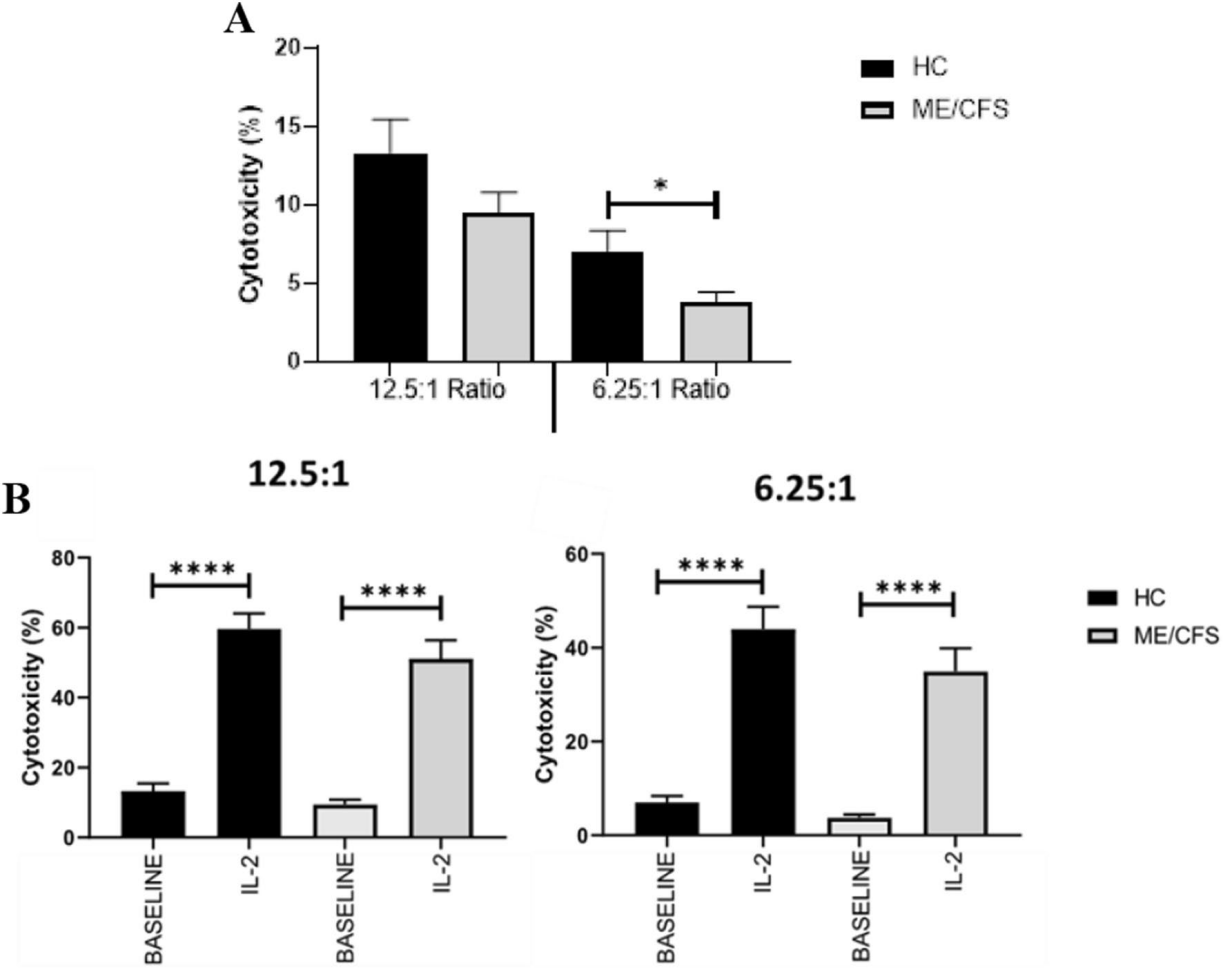
**A**. Baseline NK cell cytotoxicity. **B**. Enhanced NK cell cytotoxicity following overnight IL-2 stimulation compared with baseline. Bar graphs represent percentage of K562 cell death for all effector:target ratios; 12.5:1 and 6.25:1. Data presented as mean ± SEM and *p < 0.05; ***p < 0.0001. HC, healthy controls; ME/CFS, Myalgic encephalomyelitis/chronic fatigue syndrome; IL-2, interleukin-2

There was a significant decrease in co-localisation of TRPM3 with actin in control NK cells in ME/CFS patients compared with HC (PCC, p = 0.0002: MOC, p =  < 0.0001) (Fig. 5A). There was a significant decrease in co-localisation values for TRPM3 with PIP_2_ in control NK cells in ME/CFS patients compared with HC (PCC, p = 0.0018: MOC, p = 0.0021) (Fig. 5B). There was a significant increase in co-localisation values for TRPM3 with PIP_2_ in PregS (30 µM) + ononetin (3 µM) treated NK cells in ME/CFS patients compared with HC (PCC, p = 0.0408) (Fig. 6). No other significance was reported between groups.

### Impaired baseline NK cell cytotoxicity in ME/CFS patients enhanced following IL-2 stimulation

Cytotoxic activity was determined using flow cytometry to assess NK cell lysis of tumour target K562 cell line for both HC and ME/CFS patients. A significant decrease in cytotoxicity was reported at 6.25:1 ratio in ME/CFS patients compared with HC (p = 0.0362) (Fig. 6A). No other significance in cytotoxicity were reported between HC and ME/CFS patients. The effect of 24-h IL-2 (20 IU) stimulation on NK cell cytotoxicity was determined using flow cytometry (Fig. 6B). There was a significant increase in NK cell cytotoxicity in HC and ME/CFS patients following overnight stimulation with IL-2 across all E:T ratios.

### NK cell cytotoxicity modulation with PregS and ononetin

**Fig. 7:**
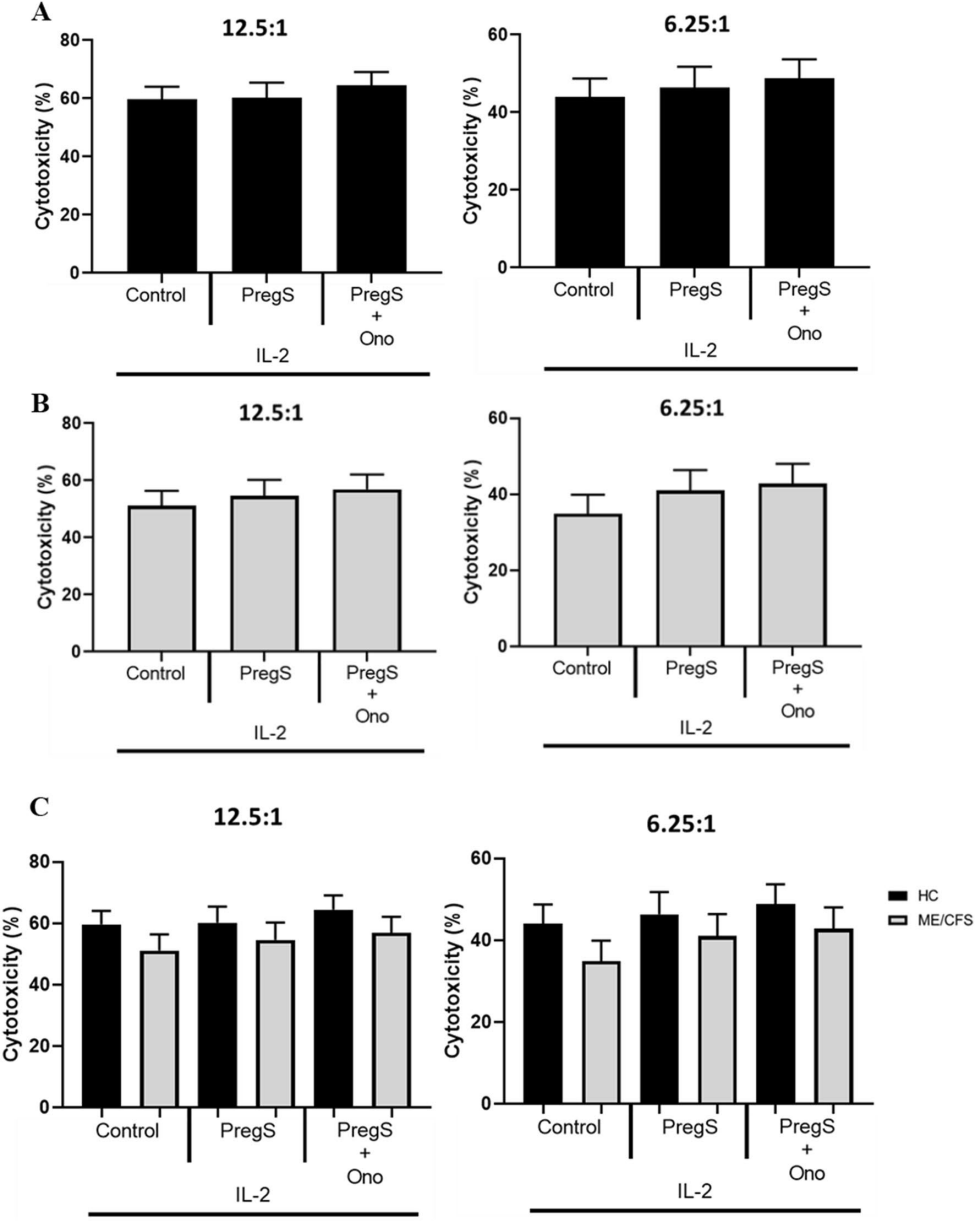
**A** NK cell cytotoxicity in HC following treatment with PregS and PregS + Ono. **B** NK cell cytotoxicity in ME/CFS patients following overnight treatment with PregS and PregS + Ono. **C** Mann Whitney U test comparison NK cell cytotoxicity between ME/CFS patients and HC following overnight treatment with PregS and PregS + Ono. Bar graphs represent percentage of K562 cell death for effector:target ratios 12.5:1 and 6.25:1. Data presented as mean ± SEM. HC, healthy control; ME/CFS, Myalgic encephalomyelitis/chronic fatigue syndrome; IL-2, interleukin-2; PregS, pregnenolone sulfate; Ono, ononetin

The effect of PregS (30 µM) and PregS + ononetin (3 µM) on NK cell cytotoxicity was determined using flow cytometry in HC. Stimulation of NK cells using IL-2 was done in conjunction with pharmacological agents. There were no significant differences in NK cell cytotoxicity between the control (IL-2) alone and pharmacological agents in HC across all E:T ratios (Fig. 7A). The effect of PregS (30 µM) and PregS + ononetin (3 µM) on NK cell cytotoxicity was determined using flow cytometry in ME/CFS patients. Stimulation of NK cells using IL-2 (20 IU) was done in conjunction with pharmacological agents. There were no significant differences in NK cell cytotoxicity between the control (IL-2) alone and pharmacological agents in ME/CFS patients across all E:T ratios (Fig. 7B). Non-parametric T test was used to determine significance in NK cell cytotoxicity following TRPM3 treatment and IL-2 (20 IU) stimulation in HC and ME/CFS patients. While a decrease in cytotoxicity was noted for ME/CFS patients, this did not reach significance (Fig. 7C).

## Discussion

In recent years, research has demonstrated that TRPM3 channel dysfunction and impaired Ca^2+^ mobilisation in the pathology of ME/CFS using a validated NK cell model [5, 7, 12–14, 41]. In the present study, we used a novel immunofluorescent technique to determine co-localisation of TRPM3 with PIP_2_ in NK cells following overnight treatment with PregS and ononetin. For the first time, we report significant changes in co-localisation both within participant groups and between groups. To the authors knowledge, this investigation is novel as it is the first to report on the co-localisation of TRPM3 with PIP_2_ in NK cells in HC and ME/CFS patients. Flow cytometry was used to determine NK cell cytotoxicity in HC and ME/CFS patients prior to and following overnight incubation with IL-2 with PregS and ononetin. This current investigation reports on the potential crosstalk between IL-2-dependent and TRPM3-dependent intracellular signalling pathways may be implicated in impaired NK cell cytotoxic function in ME/CFS patients.

Surface expression of TRPM3 on NK cells was first identified using flow cytometry in both HC and ME/CFS patients whereby changes in TRPM3 expression were reported within and between participant groups following stimulation with PregS [7]. In this current investigation we reported significant changes in co-localisation of TRPM3 with actin of NK cells in HC and ME/CFS patients following overnight treatment with PregS and/or ononetin. Changes in surface ion channel expression is dependent on homeostatic regulation where diminished channel insertion in the plasma membrane is linked to loss of amino acid residues which encodes a region known as the indispensable channel function, a region required for TRPM3 function [47]. Therefore, TRPM3 isoforms may utilise different mechanisms to insert into the plasma membrane [48]. Mechanistically, protein-to-protein interactions ensure the efficient delivery of vesicle cargo to the membrane [49]. Vesicle trafficking relies on a superfamily of proteins present in all organelles referred to as SNARE proteins (soluble N-ethylmaleimide-sensitive-factor accessory protein-receptor) [49]. The regulation of SNARE-mediate fusion is reliant on a significant increase in Ca^2+^. Thus, translocation of TRP channels is controlled by increases in Ca^2+^ influx.

Previous investigations have reported no significant differences in TRPM3 expression following treatment with PregS on NK cells of ME/CFS patients [12]. A finding consistent with this current investigation. Moreover, previous findings have shown significantly reduced TRPM3 expression on CD56^Bright^ NK cells of ME/CFS patients compared with HC using flow cytometry [7]. We too reported a significant reduction in co-localisation of TRPM3 with actin in ME/CFS patients compared with HC under control conditions using a novel immunofluorescent technique. Future investigations may aim to determine whether the loss of Ca^2+^ mobilisation in ME/CFS patients consequentially leads to a loss in protein translocation. Interestingly, CaM may participate in channel protein trafficking in TRP channels as reports have demonstrated that a loss in CaM binding sites reduces surface expression of TRPC3 [50]. Overall, mechanisms involved in assembly and trafficking of TRP channels control their plasma membrane expression and impact their function and/or regulation. It is noteworthy that we are unable to determine if loss of co-localisation is attributable to reduced TRPM3 expression on NK cells without further investigation. However, we can hypothesise that poor Ca^2+^ signalling may reflect changes in TRPM3 expression that contribute to the pathomechanism of ME/CFS.

While TRPM3 regulation is dependent on external stimuli and changes in intracellular Ca^2+^ levels, phosphatidylinositol (PPI) proteins represent an important mechanism in TRP channel modulation and downstream stimulation [23]. Specifically, the CaM binding site of TRPM3 is believed to convey intracellular signals through PIP_2_ resulting in activation of protein kinases, a rise in intracellular Ca^2+^ and regulation of cell function [51–53]. This current manuscript is novel as we investigated, for the first time, the co-localisation of TRPM3 with PIP_2_. The levels of PIP_2_ in the cell can be rapidly altered by the activity of PPI-directed kinases and phosphatases resulting in the modulation of TRP channel activity [54, 55]. PLC activation catalyses the degradation of PIP_2_ resulting in the generation of DAG and IP_3_. It has been shown that activation of PLC regulates TRPM3 by limiting the availability of PIP_2_ [23]. This signalling cascade modulates many intracellular pathways to regulate recruitment and activation of signalling proteins, reorganisation of cytoskeleton, intracellular Ca^2+^ mobilisation and activation of cell effector functions. Potentially, cell variations in the level of regulatory proteins may explain these effects on TRP channel activity [56].

This current project hypothesised that changes in PIP_2_ co-localisation may contribute to TRPM3 channel dysfunction in ME/CFS patients. PIP_2_ plays an essential role in NK cell cytotoxicity through store-operated Ca^2+^ entry pathways that ultimately stimulates the phosphorylation and activation of protein kinases, microtubule rearrangement for cytolytic granule polarisation, release of lytic proteins, formation of the perforin pore and granzyme-dependent target cell death [29, 57, 58]. Therefore, changes in PIP_2_ levels can either impede or increase lymphocyte function. Interestingly, in the absence of Ca^2+^, PIP_2_ can undergo conformational changes and act as an anchor required for plasma membrane rearrangement during immune effector functions [24]. Previous investigations have suggested that the presence of PIP_2_ is required to enhance TRPM3 channel activity [23, 59]. It is interesting that ononetin, a TRPM3 antagonist, resulted in a significant increase in co-localisation with PIP_2_. However, ononetin is a poorly characterised compound and is known to inhibit not only TRPM3, but other TRP channels as well as confer antioxidant properties [22, 60]. Therefore, changes in PIP_2_ levels in ME/CFS patients after ononetin may be due to uncharacterised off-target effects. Moreover, pharmacological tools may not effectively enhance channel activity, potentially due to reduced channel sensitivity which has been observed in ME/CFS patients using patch clamp experiments [5, 13]. Investigating the effect of PIP_2_ on channel expression and NK cell function in ME/CFS patients may provide insight into any potential feedback mechanism that offers protection to impaired cells.

While the pathology of ME/CFS is relatively unknown, impaired NK cell cytotoxicity is consistently documented [41], a result we also report in this current investigation. Stimulation of NK cells with IL-2 is known to greatly enhance cytotoxicity compared with naïve NK cells [61, 62]. This is consistent with our findings as we report a significant increase in NK cell cytotoxicity following overnight stimulation with IL-2 in both HC and ME/CFS patients. Thus, this current investigation is the first to report that IL-2 stimulation may improve NK cell cytotoxicity in vitro in ME/CFS patients. However, we cannot conclude on the effect of IL-2 on NK cell cytotoxicity in vivo without further investigation. Moreover, as IL-2 stimulation efficiently primes NK cell function in both HC and ME/CFS patients, the effect of PregS and ononetin may be lessened. Regulation of NK cell cytotoxic function relies on equilibrium between multiple synergistic or even opposing pathways [63–65]. The expression and signalling pathways of TRPM3 channels and IL-2 receptors (IL-2R) have been elucidated [12, 33]. An investigation by Ross et al*.* reported that IL-2 dependent Jak activity and the control of PI3K-protein kinase B (PKB) pathways are important for immune function through the integration of signalling pathways and protein phosphorylation [66]. Therefore, the priming effect of IL-2 on NK cell cytotoxicity is attributable to the activation and crosstalk between multiple signalling pathways including Ras-MAPK, JAK-STAT and PI3K-PKB [66–70] which is believed to be facilitated by PPIs [71]. Thus, there is an intracellular bridge between IL-2 and TRPM3 signalling proteins.

A previous investigation reported a significant reduction in the phosphorylation of ERK1/2 and MEK1/2 in NK cells of ME/CFS patients compared with HC [72]. Moreover, intracellular pathways which provide energy in response to cytokine activation are reduced in ME/CFS patients [73, 74] and as a consequence, this is believed to contribute to changes in NK cell degranulation and release of lytic proteins resulting in impaired cytotoxic function [40, 75–77]. Moreover, nuclear factor kappa B (NF-ĸB) is responsible for the regulation of genes encoding cytokine and cytokine receptors [78]. Aberrations in NF-ĸB have been reported in ME/CFS patients thus believed to influence cytokine expression [39, 79]. Significant reductions in T-lymphocyte production of IL-2 have been reported in ME/CFS patients [80] while serum or plasma cytokine concentration for IL-2 has been reported as significantly higher in ME/CFS patients compared with HC [81]. Increases in serum IL-2 may be a pathway which attempts to compensate for the loss of protein kinase phosphorylation and TRPM3-dependent cell dysfunction in ME/CFS patients. IL-2 and TRPM3-dependent pathways are reliant on Ca^2+^ to effectively activate cell function through recruitment and activation of signalling proteins [82, 83]. As Ca^2+^ mobilisation is impaired in ME/CFS patients, this may further impair IL-2 ability to enhance function. The activation of TRPM3 and IL-2R provides an example for the integration of separate signalling pathways through a phenomenon described as “crosstalk”. Thus, under normal conditions, pathways involved in TRPM3 and IL-2 signalling may work synergistically to achieve function in NK cells. To the authors knowledge, this current investigation is the first to demonstrate a possible interaction between IL-2 and TRPM3 pathways in isolated NK cells. Further investigation into IL-2-dependent and TRPM3-dependent expression and phosphorylation in ME/CFS patients is required.

The results in this current investigation are considered preliminary due to the small sample size. Therefore, the findings of this publication warrant further investigation with a larger cohort. It is important to note that co-localisation does not necessarily reflect TRPM3 surface expression and further investigation assessing splice variants of TRPM3 that are not activated by PregS may be required for further TRMP3-colocalisation characterisation [10]. Moreover, further investigation using electrophysiology and western blot techniques would provide further insight into the potential relationship between IL-2, TRPM3 and PIP_2_ in the pathomechanism of ME/CFS. Investigating differences in TRPM3 expression in various severity states of ME/CFS could elucidate the involvement of TRPM3 in the pathology of ME/CFS.

## Conclusion

TRPM3 ion channels are significant contributors to Ca^2+^ signalling and sustained Ca^2+^ influx is required to drive many biological pathways: for example, NK cell cytotoxic function. Therefore, we postulate that impaired TRPM3 channel function may impede Ca^2+^ signalling in NK cells of ME/CFS patients resulting in reduced NK cell function. These findings support the use of NK cell cytotoxicity as a cellular model for continued research on impaired TRPM3 ion channel function which is believed to contribute to loss of NK cell effector function in ME/CFS patients. For the first time, we report changes in co-localisation which suggest PIP_2_-dependent TRPM3 function may be impaired in ME/CFS patients and future research could elucidate this hypothesis. Overnight stimulation with IL-2 enhanced cytotoxicity function in HC and enhanced cytotoxicity in ME/CFS patients. A crosstalk exists between IL-2 and TRPM3 intracellular signalling pathways which are dependent on Ca^2+^ influx and PIP_2_. While IL-2R responds to IL-2 binding in vitro, Ca^2+^ dysregulation and impaired intracellular signalling pathways impede NK cell function in ME/CFS patients. This crosstalk provides a potential investigative target for future research in ME/CFS pathomechanism.

## Supplementary Information

**Additional file 1: Figure S1.** NK cells were stained with CD3 APH-H7 (5µl/test) and CD56 BV650 (20µl/test) monoclonal antibodies (Becton Dickinson [BD] Biosciences, San Jose, CA, USA). Cells were acquired at 10,000 events using the Accuri C6 flow cytometer (BD Biosciences, San Diego, CA, USA). Gating strategy is as follows: (A) lymphocytes were gated based of SSC and FSC. (B) CD3 negative population was gated from selected lymphocyte population. Gating was determined using isotype controls. (C) NK cell purity was determined based on CD56 positive cells using the CD3 negative population. **Figure S2.** Bar graphs representing NK cell purity (%) determined using flow cytometry methods. Data presented as mean ± SEM. **Figure S3.** NK cell cytotoxicity was used to determine EC50 of PregS. **Figure S4.** NK cell cytotoxicity was used to determine IC50 of ononetin. **Figure S5.** (A) NK cells and K562 cells were gated based on SSC and FSC. (B) NK cells were labelled with Paul Karl Horan (PKH)-26 (3.5µl/test) and are presented in the lower right quadrant. K562 cells were PKH negative and are presented in the upper left quadrant. (C) K562 cell death was determined by selecting PKH- cells above 200,000 SSC. Live cells were 7-AAD and Annexin V negative (lower left quadrant). Cells in early apoptosis were negative for 7-AAD, but positive for Annexin V (upper left quadrant). Cells in late apoptosis were positive for both Annexin V and 7-AAD (upper right quadrant). Necrotic or dead cells were positive for 7-AAD and negative for Annexin V (lower right quadrant). Cytotoxicity (%) was determined using the below equation: ${\text{Cytotoxicity }}\left( {\text{\% }} \right) = { }\frac{{({\text{early stage apoptosis}} + {\text{late stage apoptosis}} + {\text{necrotic cells}}}}{{{\text{All K}}562{\text{ cell events}}}} \times 100.$

## Data Availability

Datasets analysed and/or generated during the current study are not publicly available due to confidentiality agreements but are available from the corresponding author upon reasonable request.

## Abbreviations

μORs: Mu-opioid receptors
7-AAD: 7-Amino-actinomycin
AP-1: Activator protein-1
BMI: Body mass index
BD: Becton Dickinson
Ca^2+^: Calcium
[Ca^2+^]_I_: Intercellular calcium
CaM: Calmodulin
CCC: Canadian Consensus Criteria
CFS: Chronic fatigue syndrome
DAG: Diacylglycerol
EDTA: Ethylenediaminetetraacetic acid
ER: Endoplasmic reticulum
FBS: Fetal bovine serum
FITC: Fluorescein isothiocyanate
HC: Healthy control
IL-2: Interleukin 2
ICC: International Consensus Criteria
IP_3_: Inositol 1,4,5-trisphosphate
IP_3_R: Inositol 1,4,5-trisphosphate receptor
JAK: Janus tyrosine kinase
MAPK: Mitogen activated protein kinase
ME: Myalgic encephalomyelitis
Mg^2+^: Magnesium
Mn^2+^: Manganese
Na^2+^: Sodium
NK: Natural killer
PI3K: Phosphoinositide 3-kinase
PPI: Phosphatidylinositol
PIP2: Phosphatidylinositol 4,5-bisphosphate
PKB: Protein kinase B
PKH-26: Paul Karl horan-26
PLC: Phospholipase C
PregS: Pregnenolone sulfate
RPMI: Roswell park memorial institute medium
STAT: Signal transducer and activator of transcription
SF-36: 36 Item short form survey
SNARE: Soluble *N*-ethylmaleimide-sensitive-factor accessory protein-receptor
SNPs: Single nucleotide polymorphism
TRPM3: Transient receptor potential melastatin 3
WHO DAS: World health organization disability assessment schedule
