# Supplementary material for: The effect of IL-2 stimulation and treatment of TRPM3 on channel co-localisation with PIP2 and NK cell function in myalgic encephalomyelitis/chronic fatigue syndrome patients
Source: J Transl Med. 2021 Jul 15;19:306. doi: 10.1186/s12967-021-02974-4 (PMC8281618; doi:10.1186/s12967-021-02974-4)
Supplement: Supplementary file 1 — Additional file 1: Figure S1. NK cells were stained with CD3 APH-H7 (5µl/test) and CD56 BV650 (20µl/test) monoclonal antibodies (Becton Dickinson [BD] Biosciences, San Jose, CA, USA). Cells were acquired at 10,000 events using the Accuri C6 flow cytometer (BD Biosciences, San Diego, CA, USA). Gating strategy is as follows: (A) lymphocytes were gated based of SSC and FSC. (B) CD3 negative population was gated from selected lymphocyte population. Gating was determined using isotype controls. (C) NK cell purity was determined based on CD56 positive cells using the CD3 negative population. Figure S2. Bar graphs representing NK cell purity (%) determined using flow cytometry methods. Data presented as mean ± SEM. Figure S3. NK cell cytotoxicity was used to determine EC50 of PregS. Figure S4. NK cell cytotoxicity was used to determine IC50 of ononetin. Figure S5. (A) NK cells and K562 cells were gated based on SSC and FSC. (B) NK cells were labelled with Paul Karl Horan (PKH)-26 (3.5µl/test) and are presented in the lower right quadrant. K562 cells were PKH negative and are presented in the upper left quadrant. (C) K562 cell death was determined by selecting PKH- cells above 200,000 SSC. Live cells were 7-AAD and Annexin V negative (lower left quadrant). Cells in early apoptosis were negative for 7-AAD, but positive for Annexin V (upper left quadrant). Cells in late apoptosis were positive for both Annexin V and 7-AAD (upper right quadrant). Necrotic or dead cells were positive for 7-AAD and negative for Annexin V (lower right quadrant). Cytotoxicity (%) was determined using the below equation: [file 12967_2021_2974_MOESM1_ESM.docx]

# **Additional file**

**Figure S1: NK Cell Purity**

**
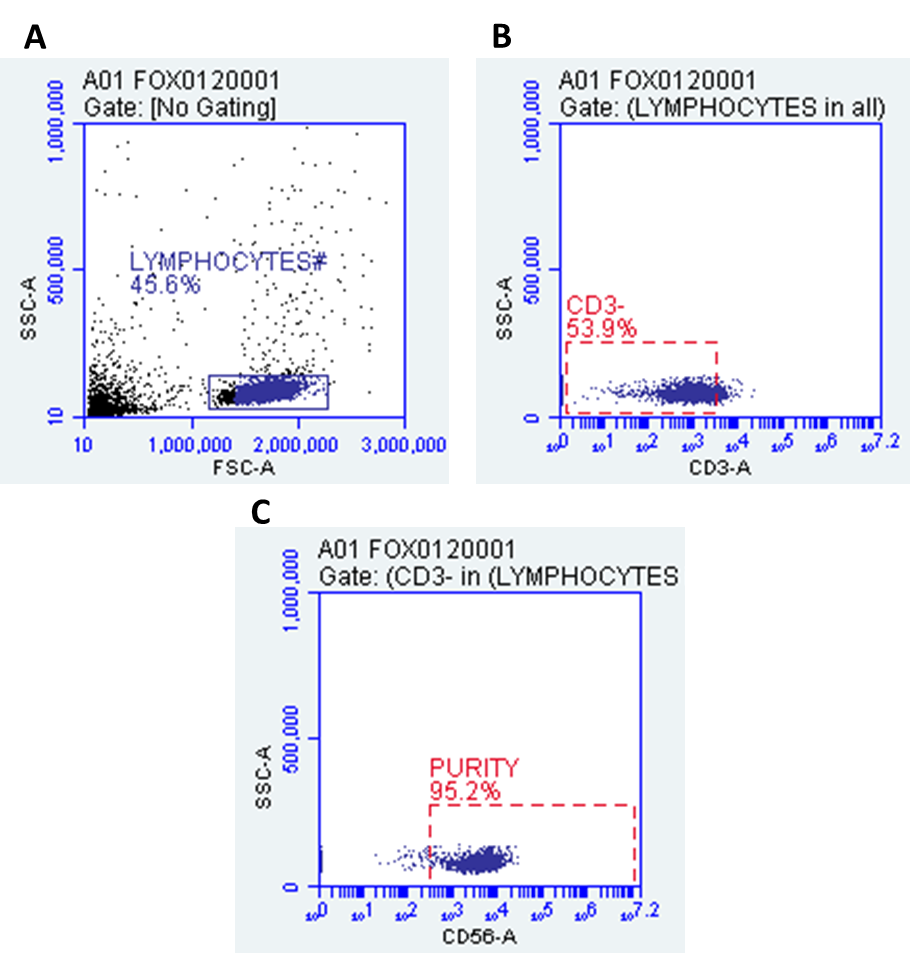
**

**Figure S1.** NK cells were stained with CD3 APH-H7 (5µl/test) and CD56 BV650 (20µl/test) monoclonal antibodies (Becton Dickinson [BD] Biosciences, San Jose, CA, USA). Cells were acquired at 10,000 events using the Accuri C6 flow cytometer (BD Biosciences, San Diego, CA, USA). Gating strategy is as follows: (A) lymphocytes were gated based of SSC and FSC. (B) CD3 negative population was gated from selected lymphocyte population. Gating was determined using isotype controls. (C) NK cell purity was determined based on CD56 positive cells using the CD3 negative population.

**Figure S2: NK Cell Purity**

**Figure S2.** Bar graphs representing NK cell purity (%) determined using flow cytometry methods. Data presented as mean ± SEM.

**Figure S3. PregS Dose Response**

**
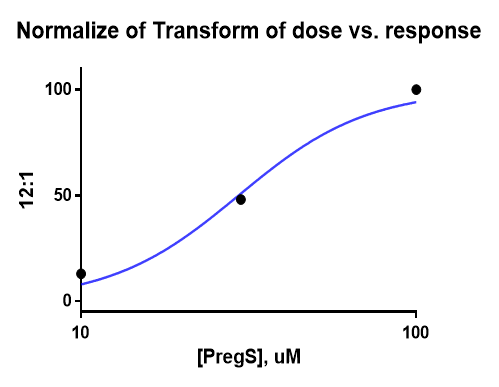
**

**Figure S3.** NK cell cytotoxicity was used to determine EC50 of PregS

**Figure S4: Ononetin Dose Response**


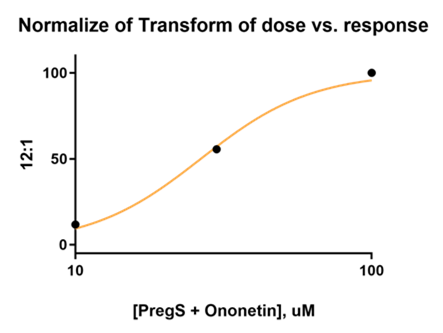


**Figure S4.** NK cell cytotoxicity was used to determine IC50 of ononetin.

**Figure S5. NK Cell Cytotoxicity Gating**

**
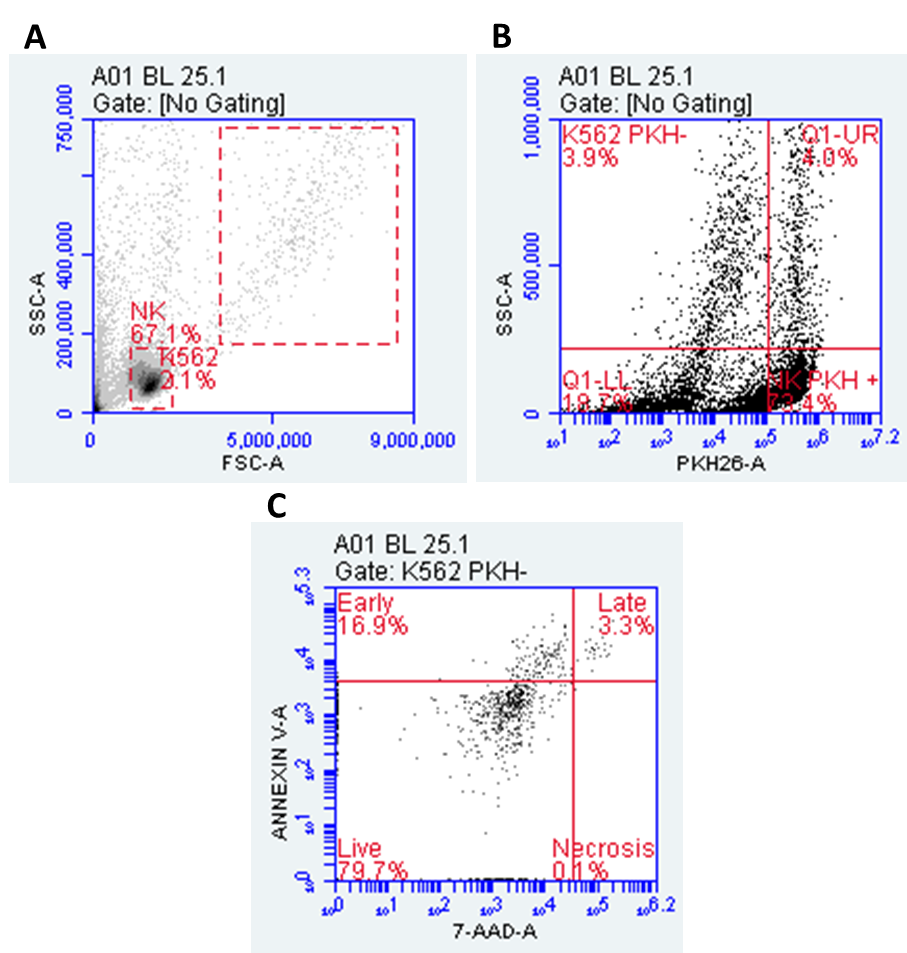
**

**Figure S5.** (A) NK cells and K562 cells were gated based on SSC and FSC. (B) NK cells were labelled with Paul Karl Horan (PKH)-26 (3.5µl/test) and are presented in the lower right quadrant. K562 cells were PKH negative and are presented in the upper left quadrant. (C) K562 cell death was determined by selecting PKH- cells above 200,000 SSC. Live cells were 7-AAD and Annexin V negative (lower left quadrant). Cells in early apoptosis were negative for 7-AAD, but positive for Annexin V (upper left quadrant). Cells in late apoptosis were positive for both Annexin V and 7-AAD (upper right quadrant). Necrotic or dead cells were positive for 7-AAD and negative for Annexin V (lower right quadrant). Cytotoxicity (%) was determined using the below equation:

$$\mathrm{Cytotoxicity}\left( \% \right)= \frac{(early stage apoptosis+late stage apoptosis+necrotic cells}{All K562 cell events}\times100$$
